# Prevalence and associated risk factors for *Lawsonia intracellularis* infection in farmed rabbits: A serological and molecular cross-sectional study in South Korea

**DOI:** 10.3389/fvets.2023.1058113

**Published:** 2023-02-09

**Authors:** Jung-Yong Yeh

**Affiliations:** ^1^Department of Life Sciences, College of Life Sciences and Bioengineering, Incheon National University, Incheon, Republic of Korea; ^2^Research Institute for New Drug Development, Incheon National University, Incheon, Republic of Korea; ^3^Convergence Research Center for Insect Vectors, Incheon National University, Incheon, Republic of Korea; ^4^KU Center for Animal Blood Medical Science, College of Veterinary Medicine, Konkuk University, Seoul, Republic of Korea

**Keywords:** antibody, epidemiology, IPMA, proliferative enteropathy, qPCR, shedding

## Abstract

*Lawsonia intracellularis* is the etiological agent of proliferative enteropathy, which is globally considered an important enteric disease in pigs and horses. Experimental studies suggest that the organism spreads by subclinical infection of many animals, including rabbits. Despite the importance of rabbits in the epidemiology of *L. intracellularis*, the extent of exposure to *L. intracellularis* in the rabbit population is poorly defined and remains unclear. The objective of this cross-sectional study was to investigate the seroprevalence and shedding of *L. intracellularis* in farmed rabbits. Furthermore, we aimed to identify risk factors associated with seropositivity. Sera from the rabbits were used to measure *L. intracellularis*-specific antibodies by immunoperoxidase monolayer assay, and rectal swabs were used to detect *L. intracellularis* DNA using a real-time PCR assay. Antibodies against *L. intracellularis* were detected in 12.3% of farms (20/163) and 6.3% of rabbits (49/774). *Lawsonia intracellularis* DNA in rectal swabs was detected in 3.8% of farms (6/156) and 1.2% of rabbits (8/667). The risk factor analysis showed that the presence of pigs or horses on the farm or the neighboring farm was associated with an increase in the risk of seropositivity (*p* < 0.05). We observed significantly increased odds of positivity for *L. intracellularis* in rabbits with a history of digestive trouble (diarrhea) on the farm during the 3 months before the samples were obtained (*p* < 0.05). Collectively, these findings demonstrated that *L. intracellularis* infection was evident among farmed rabbits and that rabbits might serve as an important reservoir for *L. intracellularis* epidemiology.

## 1. Introduction

*Lawsonia intracellularis* is a microaerophilic intracellular bacterium that infects the small and also large intestine in pigs and other animals, including hamsters and horses (1–5). The disease caused by *L. intracellularis* is characterized by cell proliferation, hemorrhage, necrosis, or any combination commonly referred to as “ileitis” or “proliferative enteropathy.” The infection is globally considered one of the important enteric diseases in pigs and horses and is known as porcine proliferative enteropathy and as equine proliferative enteropathy based on the corresponding host (6–9).

*Lawsonia intracellularis* has been associated with the colonization of enterocytes in a wide range of hosts, including rabbits, hamsters, rats, guinea pigs, swine, sheep, horses, white-tailed deer (*Odocoileus virginianus*), dogs, artic foxes (*Alopex logopus*), ferrets, ostriches (*Struthio camelus*), and rhesus macaques (10–13). The epidemiology of *L. intracellularis* is not fully known, but experimental studies suggest that the organism is spread by subclinical infection of a wide variety of animal species, including rabbits (14–17).

It is now well-known that *L. intracellularis* can infect rabbits and induce conditions referred to as acute typhlitis, histiocytic enteritis, enterocolitis, and proliferative enteropathy (11, 18–21). Despite the importance of rabbit species in *L. intracellularis* epidemiology, the extent of exposure to *L. intracellularis* in the rabbit population is poorly defined and remains unclear. The lack of baseline data on the prevalence of *L. intracellularis* in rabbits has resulted in a poor overall understanding of the epidemiology of proliferative enteropathy.

The objective of this cross-sectional study was to investigate the prevalence of antibodies to and the shedding of *L. intracellularis* in farmed rabbits in South Korea. Furthermore, we aimed to identify risk factors associated with seropositivity, including breed, sex, age, rearing type, type of rabbitries, feed type, presence of pigs or horses on the farm or the neighboring farm, and history of digestive trouble (diarrhea) on the farm.

## 2. Method

### 2.1. Rabbit farms and data collection

To obtain the serum and rectal swab samples for this study, farm selection and blood sampling were conducted in close collaboration with local veterinary practitioners and/or government veterinary officers. The study was carried out between March 2020 and February 2022, and we obtained information by carrying out farm visits as a part of veterinary practice, routine health appraisal, and consulting activities on rabbit farms. Informed consent was requested from individuals on every participating farm. Individual information about the breed, sex, age, rearing type (enclosed, outdoor, or mixed enclosed and outdoor), type of rabbitries [cage, fence (or free range)], or mixed [cage and fence (or free range)], feed type, presence of pigs or horses within the farm or the neighboring farm (within a 0.5 km radius), and history of digestive trouble (diarrhea) in the farm during the 3 months before the samplings was gathered through a detailed survey.

A true prevalence of 50% was assumed due to the absence of available previous data on prevalence of *L. intracellularis* infection among rabbits. The determined sample size needed was calculated as 28 for an unknown population size with a desired precision of 20%, a 95% confidence level and a test assumed with 95% sensitivity and 99% specificity (22, 23). A sample size of animals per rabbit farm was determined using USDA animal sample calculator (24).

### 2.2. Serum samples and rectal swabs

At each farm, blood samples and rectal swabs were taken randomly. Whole blood samples were collected from auricular veins and centrifuged to obtain sera for serological tests. The sera separated from the blood samples were stored at −80°C prior to use. Rectal swabs were collected using sterile cotton-tipped swabs inserted ~1 cm into the rectum and gently rotated to collect fecal material from the rectal wall. Swabs were retracted and placed in sterile conical tubes, the shaft was cut, and the tubes were closed and frozen at −20°C until shipped to the laboratory for DNA extraction. Rectal swabs that arrived at the laboratory were kept refrigerated at 4 °C prior to processing for nucleic acid purification within 48 h of collection. Samples with insufficient volume or poor quality were excluded from the analysis. Samples that were missing information needed for the risk analysis of this study were also excluded. A total of 774 serum and 667 rectal swab samples collected from 163 rabbit herds nationwide inland in South Korea were used in this study.

### 2.3. Serology

Sera from the rabbits were used to detect *L. intracellularis*-specific antibodies by immunoperoxidase monolayer assay (IPMA). The cultivation of *L. intracellularis* and serology using the IPMA technique were performed as described previously (25–27). The pathogenic isolate PHE/KK421 (Korean Collection for Type Cultures 10686BP, Daejeon, South Korea) was used to infect murine fibroblast-like McCoy cells (American Type Culture Collection CRL 1696, VA, USA). Briefly, a *L. intracellularis* culture plate was incubated with sera diluted at 1:60 in phosphate-buffered saline (PBS) for 30 min at 37°C and washed five times with PBS (pH 7.2). Sera collected from experimentally infected rabbits served as positive controls. Peroxidase-labeled anti-rabbit IgG antibody (Bethyl Laboratories, Montgomery, TX, USA) was diluted 1:500 in 2% bovine serum albumin and 0.08% Tween 80 in PBS and then applied at a concentration of 50 μl/well. The plate was incubated for 45 min at 37°C. The plate was washed again, and chromogen (3-amino-9-ethyl-carbazole, Dako Corporation, CA, USA) solution was added to each well. Then, the plate was incubated at room temperature for 20 min. The plate was washed with distilled water three times, allowed to dry, and examined using a BX50 microscope (Olympus, Tokyo, Japan). Positive samples exhibited red-labeled bacteria in both the cytoplasm of the infected McCoy cells and the extracellular space (28–31).

### 2.4. PCR

DNA purification was performed using a BioRobot M48 workstation apparatus (Qiagen, GmBH, Hilden, Germany) with a MagAttract DNA Mini M48 Kit (Qiagen) according to the manufacturer's recommendations. One negative extraction sample containing other bacterial cells (*Escherichia coli*) and one positive extraction sample containing *L. intracellularis* were included in each experiment to assess for contamination during the DNA extraction process. Nucleic acids were eluted in 50 μl of buffer and stored at −80°C. All purified DNA samples were assayed for the presence of the aspartate ammonia lyase (aspA) gene of *L. intracellularis* by real-time PCR. All purified DNA samples from the rectal swabs were assayed in triplicate for the presence of the *L. intracellularis* aspA gene by real-time PCR as described previously (22). This real-time TaqMan PCR assay enables the detection of a specific 104-base pair product of the aspA gene from *L. intracellularis* (GenBank accession no. AM180252).

Precautions were taken to minimize contamination during the precipitation, preamplification, and amplification steps, including performing all pipetting steps in a laminar flow cabinet and including positive (DNA from cell-grown *L. intracellularis*) and negative (*L. intracellularis*-free DNA from rectal swab samples) DNA controls. Furthermore, swabs were taken from centrifuges, laminar flow cabinets, and countertops and assayed for the *L. intracellularis* aspA gene by real-time PCR to assess potential contamination. A real-time PCR assay that targeted a universal sequence of the bacterial 16S rRNA gene was used as a quality control (i.e., efficiency of DNA purification and amplification) and as an indicator of fecal inhibition as described previously (32, 33).

### 2.5. Statistical analysis

Prevalence and Wilson's 95% confidence intervals (CIs) (34) were calculated using Epitools-Epidemiological Calculators (35). A logistic regression model was used to identify potential risk factors that were correlated with animal seropositivity. The variables in the univariable analysis were evaluated for pairwise collinearity or associations using Pearson's correlation coefficient or the chi-squared test for continuous or categorical variables, respectively. The strength of association was analyzed using odds ratios and 95% CIs. Results with a *p*-value < 0.05 were considered statistically significant. Statistical analyses in this study were performed using commercially available statistical software (SPSS Statistics for Windows, Version 25.0, IBM Corp., Armonk, NY, USA).

## 3. Results

**Figure 1:**
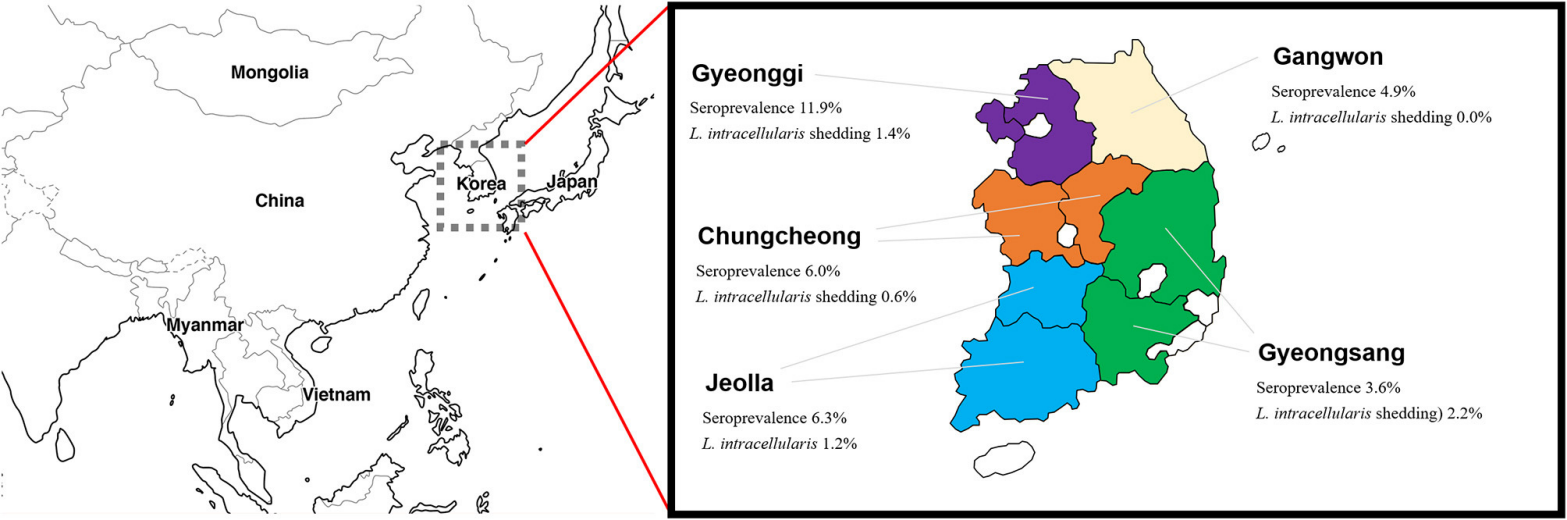
Geographical location of the provinces of South Korea and prevalence of *Lawsonia intracellularis* in farmed rabbits.

Antibodies specific to *L. intracellularis* were detected in 6.3% of farmed rabbits (49/774), as shown in Table 1 and Figure 1. *Lawsonia intracellularis* DNA in the rectal swabs was detected by PCR in 1.2% farmed rabbits (8/667). Seropositive rabbits were observed in all the provinces surveyed, and the highest seroprevalence values for *L. intracellularis* were observed in Gyeonggi Province, with rates of 11.9% (28/235, 95% CI = 8.3–17.6). Rabbits shedding *L. intracellularis* were observed in most provinces surveyed except Gangwon Province.

**Table 1:** Prevalence of antibodies to and shedding of *Lawsonia intracellularis* in farmed rabbits in South Korea.

**Province**	**Test**	**Positive**	**Negative**	**Tested**	**AP**	**TP ±95% CI**
Chungcheong	IPMA	10	157	167	6.0	2.6–10.9
	PCR	1	158	159	0.6	0.0–2.6
Gangwon	IPMA	3	58	61	4.9	0.8–14.0
	PCR	0	43	43	0.0	0.0–7.7
Gyeonggi	IPMA	28	207	235	11.9	8.3–17.6
	PCR	3	213	216	1.4	0.0–3.2
Gyeongsang	IPMA	6	163	169	3.6	0.7–7.3
	PCR	3	135	138	2.2	0.0–5.5
Jeolla	IPMA	2	140	142	1.4	0.0–4.5
	PCR	1	110	111	0.9	0.0–4.2
Total	IPMA	49	725	774	6.3	4.3–8.2
	PCR	8	659	667	1.2	0.0–1.4

AP, apparent (estimated) prevalence; TP, true prevalence; CI, confidence interval; IPMA, immunoperoxidase monolayer assay; PCR, rectal swab PCR.

In the univariate analysis (Table 2), the risk factor analysis revealed that the presence of pigs or horses on the farm or the neighboring farm (within a 0.5 km radius) was associated with an increase in the risk of seropositivity (OR = 1.480, 95% CI = 1.117–1.959, *p* < 0.05). We observed significantly increased odds of positivity for *L. intracellularis* in the rabbits with a history of digestive trouble (diarrhea) on the farm during the 3 months before the samples were acquired (OR = 1.909, 95% CI = 1.127–3.234, *p* < 0.05). Although there was slightly higher seropositivity in crossbred animals, females, animals younger than 6 months old, animals that were reared outdoors only, animals in rabbitries with a fence or that were free range, or animals that were fed concentrated feed and grass, these associations were not statistically significant (*p* > 0.05).

**Table 2:** Univariable analysis of *Lawsonia intracellularis* exposure variables relative to seropositivity outcomes in farmed rabbits in South Korea.

**Exposure variable**	**Label**	**Positive**	**Total**	**OR (95% CI)**	***P*-value**
Breed	*Oryctolagus cuniculus*				
	Crossbreed	12	149	1.199 (0.887–1.620)	0.424
	Flemish giant	14	257	1.040 (0.806–1.342)	1.000
	New Zealand white	19	282	1.084 (0.890–1.3191)	0.616
	*Chinchilla* spp. Chinchilla	4	86	–	
Sex	Male	18	319	–	
	Female	31	455	1.082 (0.866–1.351)	0.551
Age	< 6-month-old	30	420	1.138 (0.902–1.437)	0.375
	>6-month-old	19	354	–	
Rearing type	Enclosed only	16	260	–	
	Outdoor only	18	316	0.963 (0.460–1.845)	0.860
	Mixed (enclosed and outdoor)	15	198	1.129 (0.772–1.650)	0.577
Type of rabbitries	Cage	11	215	–	
	Fence (or free range)	17	259	1.119 (0.821–1.525)	0.561
	Mixed [cage and fence (or free range)]	21	300	1.136 (0.874–1.477)	0.461
Feed	Concentrated	12	234	–	
	Concentrated and grass	37	540	1.088 (0.921–1.286)	0.424
Presence of pigs or horses within the farm or the neighboring farm (within a 0.5 km radius)	No	23	484	–	
	Yes	26	290	1.480 (1.117–1.959)	0.021
Previous history of digestive trouble (diarrhea) in the farm	No	37	663	–	
	Yes	12	111	1.909 (1.127–3.234)	0.030

OR, odds ratio; CI, confidence interval.

## 4. Discussion

Rabbits have been described as a susceptible host of *L. intracellularis* and as an animal model in the field of proliferative enteropathy research (15–21, 36–38), but the prevalence and associated risk factors in the rabbit population have not been reported thus far. The results of this study showed that exposure rates to *L. intracellularis* in farmed rabbits were not as high as those in pigs or horses.

In pigs, the seroprevalence of antibodies to *L. intracellularis* was reported to be 31.6% in European countries (39), 34.7% in Brazil (40), 57% in China (41), 84.2% within pig herds in Australia (42), 26%−59% among slaughter-age pigs in the Netherlands (43), and 44%−69% at the animal level and 100% at the farm level in South Korea (44). The prevalence of *L. intracellularis* shedding in pig feces was found to be 26.5% in European countries (39), 19.9% in South Korea (45), 65.7% at the herd level in Poland (46), and 46%−57% among slaughter-age pigs in the Netherlands (43). In horses, the seroprevalence of antibodies to *L. intracellularis* was reported to be 98.8% among adult horses in Belgium (47), and Kranenburg et al. previously demonstrated that the prevalence of *L. intracellulari* antibodies in foals increased significantly from 15% before weaning to 23% after weaning; it was 89% in yearlings and 99% in horses older than 2 years (48).

It has been reported that *L. intracellularis* infections in rabbits generally progress subclinically (14). However, the routes of *L. intracellularis* infection in rabbits are unclear, and the source of *L. intracellularis* infection in rabbits remains speculative. In this study, the presence of pigs or horses on the farm or the neighboring farm (within a 0.5 km radius) was found to be one of the main factors affecting the prevalence of *L. intracellularis* infection (*p* < 0.05). Another main factor identified in the present study was a history of digestive trouble (diarrhea) on the farm during the 3 months before the samples were acquired (*p* < 0.05). We observed no significant differences regarding the prevalence of *L. intracellularis* infection when rabbits were stratified by breed, sex, age, rearing type, type of rabbitries, or feed type (*p* > 0.05).

This study may serve as a basis for future epidemiological studies on *L. intracellularis* infections. The results of the present study demonstrate that the possibility that the range of susceptible species may broaden further and incorporate more domestic animals by interspecies transmission (spillover) cannot be discounted. In view of the host range of *L. intracellularis*, lagomorphs may represent a reservoir, amplifying host, or important biological vector of *L. intracellularis* due to their large population, their short reproductive cycle, and their close contact with pigs or horses.

Collectively, the results of the present study demonstrated that *L. intracellularis* infection was evident among the farmed rabbits analyzed, in which ~one in 10 farms exhibited exposure to *L. intracellularis*. The results of this study indicated that susceptible rabbits are at risk of becoming infected with *L. intracellularis*, although an outbreak of *L. intracellularis* among farmed rabbits in South Korea has yet to be reported. These results suggest that rabbit species might serve as an important reservoir for the transmission of *L. intracellularis*, highlighting the need for closer epidemiological investigation of *L. intracellularis* infections in rabbits.

## Data availability statement

The original contributions presented in the study are included in the article/supplementary material, further inquiries can be directed to the corresponding author.

## Ethics statement

The animal study was reviewed and approved by Animal Research Ethics Committee of Incheon National University. Written informed consent was obtained from the owners for the participation of their animals in this study.

## Author contributions

J-YY conceived and designed the study, conducted the laboratory experiments, wrote the first draft, and revised the manuscript.
